# Targeting Host Tyrosine Kinase Receptor EPHA2 Signaling Affects Uropathogen Infection in Human Bladder Epithelial Cells

**DOI:** 10.3390/pathogens11101176

**Published:** 2022-10-12

**Authors:** Prema S. Prakash, Alexander Kruse, Christian Vogel, Undraga Schagdarsurengin, Florian Wagenlehner

**Affiliations:** Clinic for Urology, Pediatric Urology and Andrology, Justus-Liebig-University, 35390 Giessen, Germany

**Keywords:** uropathogens, urinary tract infections, receptor tyrosine kinases, EPHA2 inhibition, bladder epithelial cells, intracellular bacteria

## Abstract

Urinary tract infections (UTIs) affect a major proportion of the world population but have limited non-antibiotic-based therapeutic and preventative strategies against UTIs. Facultative intracellular uropathogens such as strains of uropathogenic *E. coli*, *K. pneumoniae*, *E. faecalis*, *E. cloacae* are well-known uropathogens causing UTIs. These pathogens manipulate several host-signaling pathways during infection, which contributes to recurrent UTIs and inappropriate antibiotic application. Since host cell receptor tyrosine kinases (RTKs) are critical for the entry, survival and replication of intracellular pathogens, we investigated whether different uropathogens require host EPHA2 receptors for their intracellular survival using a cell culture model of intracellular infection in human bladder epithelial cells (BECs). Infection of BECs with seven different uropathogens enhanced the expression levels and activation of EPHA2. The significance of EPHA2 signaling for uropathogen infection was investigated by silencing EPHA2 expression using RNA interference or by inhibiting the kinase activity of EPHA2 using small-molecule compounds such as dasatinib or ALW-II-41-27. Both preventive and therapeutic tyrosine kinase inhibition significantly reduced the intracellular bacterial load. Thus, our results demonstrate the involvement of host cell EPHA2 receptor during intracellular uropathogen infection of BECs, and targeting RTK activity is a viable non-antibiotic therapeutic strategy for managing recurrent UTIs.

## 1. Introduction

Uropathogens such as uropathogenic *E. coli* (UPECs) [1,2], *K. pneumoniae* [3], *E. faecalis* [4], *E. cloacae* [5] cause urinary tract infections (UTIs), of which UPECs are the major causative bacteria leading to recurrent UTIs [6,7,8,9]. Uropathogens are known to infect bladder epithelial cells (BECs) utilizing both host and bacterial factors [10] and replicate intracellularly by manipulating several host-signaling pathways involving phosphatidylinositol 4,5-bisphosphate 3-OH kinase (PI3K), Akt kinase; and mTOR complex (mTORC) 1 and 2 [10,11]; and host endocytic recycling pathway protein Rab 35 [12]. Thus, bacterial invasion of the urinary system and intracellular infection are major pathogenicity factors for uropathogens [10]. Host cell tyrosine kinase receptors (RTKs) are known to play a critical role as host factors during the infection of epithelial cells by major intracellular pathogens [13]. UPECs invade BECs by activating kinase networks in host cells, including epidermal growth factor receptors (EGFR) [11]. 

Ephrin receptors and their ligands ephrins belong to the largest known subfamily of RTKs consisting of 16 receptors and 9 ligands that engage in a multitude of activities during development and adulthood [14]. They control important functions such as embryonic axon guidance, angiogenesis, cell death, migration and differentiation during development [15]. Among the ephrin receptors, erythropoietin-producing hepatocellular receptor A2 (EPHA2) plays a critical role in oncogenic signaling and is overexpressed in many solid tumors [16]. Therefore, EPHA2 is considered an important therapeutic target for several human malignancies [17]. EPHA2 and its ligand Ephrin-A1 functions at the crosstalk between PI3K, MAPK, Src family kinases, Rho and Rac1 GTPases [18]. Further studies confirm the crosstalk between EPHA2 and EGFR signaling in cancer [19]. Ligand activation of EPHA2 or EPHA2 knockdown by small interfering RNA inhibited EGF-induced cell motility of EGFR-overexpressing human cancer cells, indicating a functional role of EPHA2 in EGFR-expressing cancer cells [19].

UPEC activates the host PI3K-Akt pathway for the invasion process in BECs [11] and reprograms the host signaling pathways for the acquisition of nutrients, defending the host immune response and by manipulating specific host signaling factors [20,21]. Several publications reported the crosstalk between EPHA2 and PI3K-Akt pathway, and silencing of the EPHA2 gene affects the activation of the PI3K-Akt pathway [18,22,23,24]. This shows the possibility that EPHA2 could act as a host cofactor facilitating the UPEC or any other urinary tract bacteria or bladder infection contributing to uropathogenicity. Moreover, several viral infections (Human papillomavirus, Kaposi’s sarcoma-associated herpesvirus, Epstein–Barr virus, Hepatitis-B-Virus and Hepatitis C virus) [25,26,27,28,29], fungal infections (*Candida albicans*, *Pneumocystis carinii*) [30,31,32], parasite infections (Plasmodium) [33], bacterial infections (*C. trachomatis*, *H. pylori,* Enteropathogenic *E. coli*, *M. tuberculosis*) [24,34,35,36] were reported to exploit host EPHA2 signaling during infection. However, it is unexplored whether the host EPHA2 receptor and the downstream signaling are critical for uropathogen intracellular survival during its infection cycle. 

Therefore, we attempted to identify whether uropathogens such as UPECs, *K. pneumoniae*, *E. faecalis* and *E. cloacae* activate the EPHA2 signaling pathway. Our data showed increased EPHA2 expression as well as activation upon bacterial infection. Further, selective gene silencing of EPHA2 using siRNAs or by using small molecules such as clinically approved EPHA2 inhibitor dasatinib or newly developed potent EPHA2 inhibitor ALW-II-41-27 strongly inhibited the uropathogen infection in human BECs. Thus, this preclinical study demonstrates the application of RTK inhibitors as a potential non-antibiotic therapeutic alternative for the treatment of recurrent UTIs. 

## 2. Materials and Methods

### 2.1. Strains Information and Antibiotics

Pathogens used in this study were acquired from the German Collection of Microorganisms and Cell Cultures (Deutsche Sammlung von Mikroorganismen und Zellkulturen, DSMZ) or the American Type Culture Collection (ATCC) or were part of our internal strain collection. They were handled according to standard procedures. Ciprofloxacin was purchased from Sigma (#17850-25G-F), and Meropenem was purchased from TCI chemicals (#M2279).

### 2.2. Cell Culture and Bacterial Infections

Human bladder epithelial cells 5637 (BECs) (ATCC No. DSMZ ACC 35) were cultured in RPMI (Thermo Fisher Scientific #21875-034, Life Technologies Europe B.V, Bleiswijk, The Netherlands) with 10% Fetal Bovine Serum (FBS) (Sigma #S0615). Human embryonic kidney cells (HEK293) were cultured in DMEM (Thermo Fisher Scientific # 41965039, Life Technologies Europe B.V, Bleiswijk, The Netherlands) with 10% FBS. Cells were grown at 37 °C and in a humidified atmosphere of 5% CO_2_. Bacteria used in this study are *E. coli* ATCC 25922, *E. coli* 1949820 (FQ-R), *E. coli* UTI89, *E. coli* CHD94, *E. coli* K-12 MG1655, *K. pneumoniae* 595 WT, *E. faecalis* 60 and *E. cloacae* ATCC BAA-2468. The fluoroquinolone-resistant strain *E. coli* 1949820 (FQ-R) was maintained by adding ciprofloxacin (0.2 µg/mL), and the carbapenem-resistant strain *E. cloacae* ATCC BAA-2468 was maintained by adding meropenem (0.063 µg/mL) for each inoculation experiment. The mentioned bacteria were routinely cultured in LB broth (Serva #48501) or on an LB agar plate at 37 °C. Liquid cultures were grown at 37 °C under static conditions overnight in a shaker incubator (IKA^®^ KS 4000 I control). Stocks were stored frozen in glycerol at −80 °C. The multiplicity of infection (MOI) was assessed by colony-forming units per ml (CFU/mL). Cells infected with bacteria were cultured in RPMI medium in the presence of 5% FBS and were grown at 37 °C with 5% CO_2_. 

### 2.3. Antibodies and Inhibitors 

EPHA2 Monoclonal Antibody (1C11A12) Catalog # 37-4400 (WB analysis for total-EPHA2) was purchased from Thermo Scientific, and mouse monoclonal β-Actin (Catalog # ab8226) was bought from Abcam. Phospho-EPHA2 (pEPHA2 Ser897) (D9A1) Catalog # 6347 and pEphA2 Tyr772 Catalog # 8244S were purchased from Cell Signaling technology. Secondary antibodies such as IRDye^®^ 800CW Goat anti-Rabbit IgG Secondary antibody (Catalog # 926-32211) and IRDye^®^ 680RD Goat anti-Mouse IgG Secondary antibody (Catalog # 926-68070) were purchased from Licor. Inhibitors for EPHA2, such as dasatinib, were bought from Cell Signaling, and ALW-II-41-27 was bought from Sigma-Aldrich. 

### 2.4. RNA Extraction and q-PCR 

RNA extraction was performed using RNeasy Mini Kit (Qiagen, Cat. No. 74104, Hilden, Germany). Five hundred nanograms of total RNA was reverse transcribed using RevertAid H Minus First Strand cDNA Synthesis Kit (Thermo Scientific, Cat. No. K1632, Vilnius, Lithuania) according to the supplier’s instructions. The cDNA synthesis was performed using the Thermal Cycler T100 from Bio-Rad. All real-time PCR (qPCR) reactions were performed on the CFX96 Touch system (CFX 96 Real-Time system + C1000 Touch thermal cycler, Bio-Rad) with the Bio-Rad CFX Manager 3.1 software. Every primer pair was blasted with the NCBI primer blast tool (http://www.ncbi.nlm.nih.gov/tools/primer-blast/ (accessed on 13 September 2022)) and purchased from Thermo Fisher Scientific/Invitrogen. For qPCR, the iQ SYBR Green Supermix from Bio-Rad (Cat. No. 1708880) and the following cycler program were used: 5 min at 95 °C, following 40 cycles of 30 s at 95 °C, 30 s at 60 °C and 30 s at 72 °C. In order to complete the program, the samples stayed at 72 °C for 5 min and finally, the melting curve was determined in the range of 55–95 °C with an increase of 0.5 °C every 5 s. The values were calculated using the delta delta ct (ΔΔC_t_) method.

***EPHA2* accession number: NM_004431.5**

Forward primer (5′ to 3′): CTGCCAGTGTCAGCATCAAC

Reverse primer (5′ to 3′): GCTGTTGGAGTCTCCCTTCT

***ß-Actin*** **accession number: NM_001101.5**

Forward primer (5′ to 3′): GATTCCTATGTGGGCGACGAG

Reverse primer (5′ to 3′): AGGTCTCAAACATGATCTGGGT

### 2.5. Intracellular Bacterial Survival Assay 

BECs (at 1 × 10^5^ seeding density) were cultured in 12 well tissue culture plates. The next day, cells were infected with bacteria at MOI, 10–15 per host cell. After 2 h incubation at 37 °C, cells were washed three times with RPMI to remove nonadherent bacteria. Cells were then incubated for 1 h with a complete RPMI medium plus 100 μg/mL of gentamicin (VWR International, LLC, Amresco, OH, USA) to kill any extracellular bacteria. Subsequently, cells were washed and further incubated in fresh medium containing gentamicin (10 μg/mL) for the entire duration of the experiment. After 30 h of infection, cells were washed three times with sterile 1x PBS. Intracellular bacteria were recovered by means of hypotonic cell lysis (with 0.1% Triton X-100 in sterile 1x PBS), as previously described [12]. Dilutions of the lysates were plated on LB agar and incubated for 24 h at 37 °C. The next day, colonies were counted for each plate.

### 2.6. siRNA and Transfection

siRNA smart pool targeting EPHA2 (siGENOME Human EPHA2, 5 nmol, M-003116-02-0005), ON-TARGETplus non-targeting control siRNAs (5 nmol, D-001810-01-05), 5x siRNA buffer (100 mL, B-002000-UB-100) and DharmaFECT 1 Transfection Reagent (0.75 mL, T-2001-02) were all purchased from Horizon discovery. Cells at 50–60% confluent were transfected with siRNA using DharmaFECT 1 with the optimum for 40 h and infected with bacteria for 30 h at 37 °C with 5% CO_2_. ON-TARGETplus non-targeting control siRNAs were used as a control. The knocked-down infected cell lysates were subjected to WB analysis or intracellular bacterial survival assay as described.

### 2.7. Inhibitor Assay 

Cells (at 1 × 10^5^ seeding density) were cultured in 12 well tissue culture plates. The next day, they were pretreated with DMSO control or dasatinib (7 µM) or ALW-II-41-27 (7 µM) for 4 h at 37 °C, followed by infection with bacteria for 30 h. The 24 h infected cells were post-treated with DMSO control or dasatinib (7 µM) or ALW-II-41-27 (7 µM) for 6 h. After 30 h of total infection, the medium was removed, and the cells were subjected to an intracellular bacterial survival assay. 

### 2.8. Western Blotting 

The medium was removed from the infected cells, and the cells were washed three times in ice-cold 1x PBS. Cells were lysed by heating at 95 °C in 1x Laemmli buffer (Bio-Rad#1610747) and resolved by 10% sodium dodecyl sulfate (SDS)–polyacrylamide gel electrophoresis. Proteins were transferred to PVDF membranes using a semi-dry transfer cell (Bio-Rad) and blocked with intercept blocking buffer. Primary antibodies (1:1000) were incubated overnight at 4 °C. Proteins were detected using secondary antibodies (1:5000) such as IRDye^®^ 800CW goat anti-rabbit IgG secondary antibody for phospho-EPHA2 and IRDye^®^ 680RD goat anti-mouse IgG secondary antibody for total-EPHA2 and β-Actin. Proteins were detected using a Bio-Rad Chemidoc imager. The signal bands were quantified by ImageJ software.

### 2.9. Statistical Analysis

Data were presented as mean ± SD from three independent experiments except for qPCR experiments, as indicated under the figure legends. All data were analyzed using GraphPad Prism 8. Statistical significance was set at *p* ≤ 0.05. Ordinary one-way ANOVA or two-way ANOVA tests with multiple comparison tests were used, as detailed in the corresponding figure legends. The degree of significance is shown using * for *p* ≤ 0.05, ** for *p* ≤ 0.01 and *** for *p* ≤ 0.001 and **** for *p* ≤ 0.0001.

## 3. Results

### 3.1. Uropathogen Infection Enhances EPHA2 Receptor Levels in Human Bladder Epithelial Cells 

**Figure 1:**
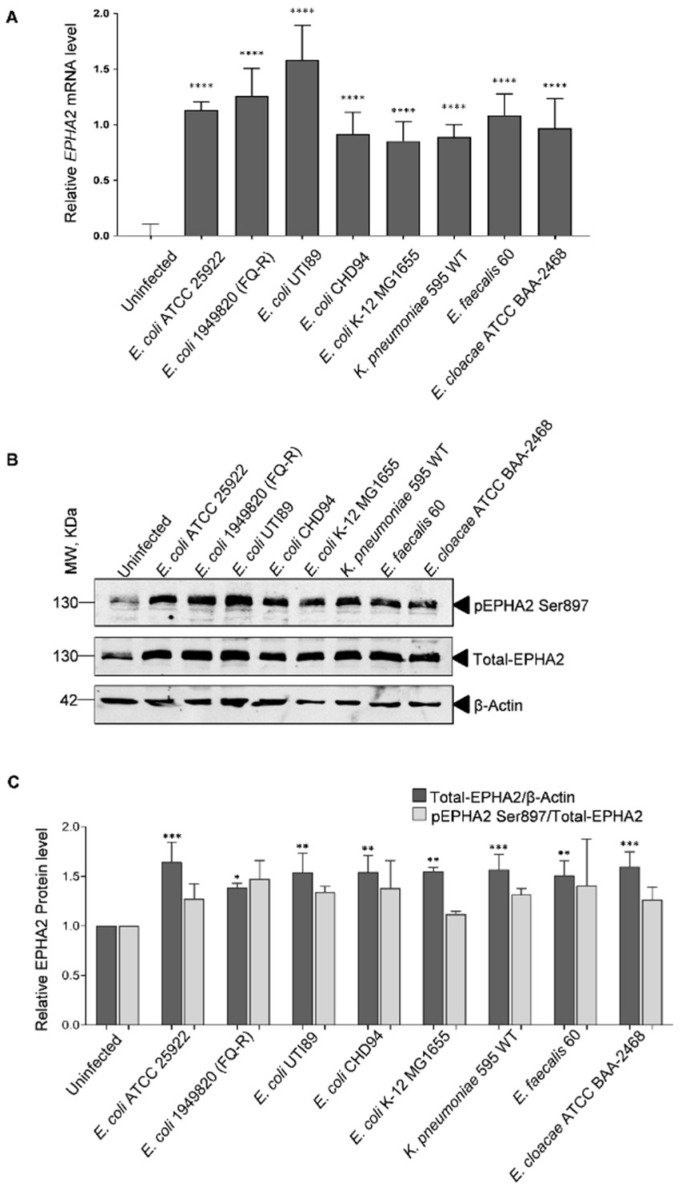
Characterization of EPHA2 receptor levels upon infection with different uropathogens. BECs were infected with different bacteria (MOI, 15–20) or maintained uninfected for 30 h at 37 °C. Medium was removed, and the cells were washed three times with 1x ice-cold PBS. Cells were then harvested and subjected to mRNA and Western blot analysis. (**A**) qPCR: *EPHA2* mRNA expression was evaluated by qPCR for uninfected and infected cell samples from four experiments after normalization to *β-Actin*. n = 4, pooled into duplicates. Statistical analysis was performed using Ordinary one-way ANOVA followed by Bonferroni multiple comparison test, **** *p* < 0.001. (**B**) Western blot membrane was first incubated with antibody against phosphorylated EPHA2 (pEPHA2 Ser897). The blot was then stripped and incubated against total-EPHA2 and β-Actin. (**C**) Band intensity for phospho-EPHA2 and total-EPHA2 was quantified by Image J analysis with respect to total-EPHA2 and β-Actin loading control. Data were presented as mean ± SD of three independent experiments. Statistical analysis was performed using Ordinary one-way ANOVA followed by Bonferroni multiple comparison test, * *p* < 0.05, ** *p* < 0.01, *** *p* < 0.001.

Several human pathogens manipulate host EPHA2 signaling during infection [24,25,26,27,28,29,30,31,32,33,34,35,36]. In order to investigate whether different uropathogens (strains of uropathogenic *E. coli*, *K. pneumoniae*, *E. faecalis*, *E. cloacae*) and non-uropathogenic *E. coli* K-12 selectively utilize EPHA2 receptors during their infection cycle, we analyzed the mRNA, total protein levels of EPHA2 receptor and their activation state 30 h post-infection in human BECs. Both mRNA and total protein levels of EPHA2 (total-EPHA2) were increased (Figure 1A–C) upon infection in human BECs. Next, we investigated and identified enhanced activation of EPHA2 as determined from the elevated phosphorylation levels at both serine 897 (pEPHA2 Ser897) and tyrosine 772 (pEPHA2 Tyr772) upon uropathogen infection (Figure 1B and Figure S1A). The elevated EPHA2 phosphorylation levels likely parallel the increased total EPHA2 levels following infection. Importantly, the increase in the total protein levels of EPHA2 and its activation state was dependent on viable bacteria, which was not observed upon infection with heat-inactivated bacteria (Figure S1B). Interestingly, infection with a non-uropathogenic *E. coli* K-12 also elevated total EPHA2 levels comparable to that of uropathogens (Figure 1A–C). Infection-induced elevated EPHA2 receptor levels confirm its potential requirement for the uropathogenic infection cycle.

### 3.2. Silencing EPHA2 Receptor Expression Prior to Uropathogen Infection Reduces Intracellular Bacterial Load

**Figure 2:**
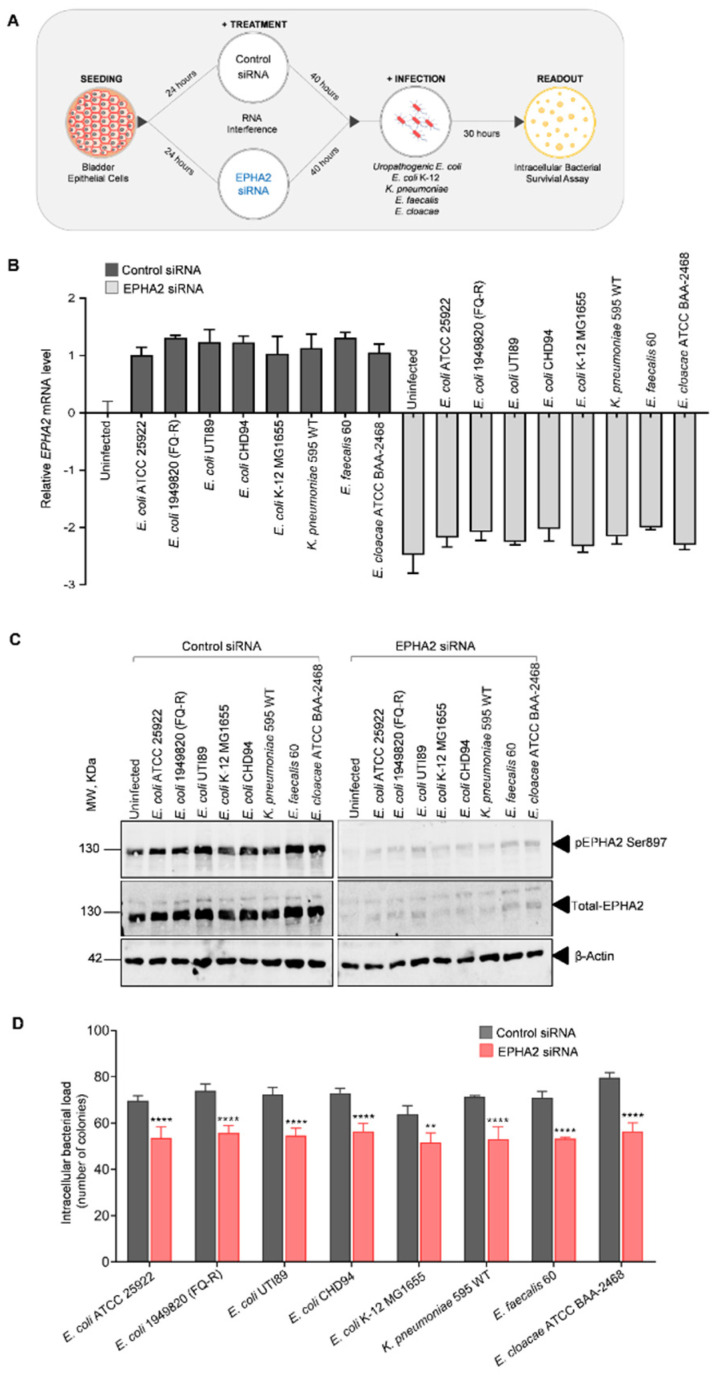
Impact of *EPHA2* RNA interference on the intracellular bacterial load. (**A**) Schematic representation of knockdown experiment performed in (**B**–**D**). BECs were transfected with *EPHA2* siRNA or control siRNA for 40 h at 37 °C. The transfected cells were left uninfected or infected with bacteria (MOI, 15–20) for 30 h at 37 °C. (**B**) qPCR: The transfection efficiency of siRNA directed against *EPHA2* was monitored using gene-specific primers. Control or knockdown of uninfected and infected cell lysates from three different experiments was pooled, and qPCR was performed. **(C)** Western blot: The cells lysates containing blot were immunoblotted—against phopho-EPHA2, total-EPHA2 and β-Actin. Protein quantity was equally loaded for each sample, and blots were placed adjacently and imaged at the same time to determine the efficiency of EPHA2 knockdown compared to control siRNA-transfected cells. (**D**) Transient knockdown of *EPHA2* using siRNA compared to the control affects uropathogens infectivity significantly as determined by colony counting. Transfected cells subjected to infection and respective uninfected controls were washed three times with 1x PBS, and intracellular bacterial survival assay was performed. Data were presented as mean ± SD of three independent experiments normalized to control siRNA. Statistical analysis was performed using two-way ANOVA followed by Bonferroni multiple comparison test, ** *p* < 0.01, **** *p* < 0.0001.

Since we observed augmented expression and activation of EPHA2 in BECs infected with uropathogens, we next questioned whether the downregulation of EPHA2 affects infection and colony formation. Therefore, using short interfering RNA (siRNA) mediated gene-specific silencing, EPHA2 protein levels were downregulated transiently in BECs. These cells were further infected with bacteria for 30 h (Figure 2A). The knockdown efficiency of EPHA2 was confirmed by qPCR and Western blotting, as shown in Figure 2B,C. Intracellular bacterial survival assay was performed by hypotonic lysing of the infected cells and plated onto LB agar plates to determine the colony numbers, which represent the intracellular bacterial load as previously shown [11,12]. The number of intracellular bacteria (the number of colonies) in BECs following infection of either uropathogenic *E. coli* strains or other uropathogens (*K. pneumoniae*, *E. faecalis*, *E. cloacae*) is significantly reduced (*p* < 0.0001) in BECs depleted of EPHA2 receptor when compared to the cells treated with non-targeting siRNAs (Figure 2D). The impact of EPHA2 depletion on the reduction in intracellular bacterial load in BECs infected with uropathogens was comparable to the outcome seen with non-uropathogenic *E. coli* K-12. This confirms that the EPHA2 receptor is commonly involved in the regulation of intracellular bacterial load, irrespective of the *E. coli* strain (non-uropathogenic or uropathogenic) and substantiates that the levels of EPHA2 receptor may effectively determine the outcome of intracellular bacterial survival in human BECs. 

### 3.3. Preventive and Therapeutic Pharmacological Inhibition of EPHA2 Kinase Activity Reduces Uropathogen Infection

In order to identify whether the kinase activity of EPHA2 is required for uropathogen infection, we utilized a clinically approved small molecule inhibitor of EPHA2 tyrosine kinase called dasatinib. This multi-kinase drug also inhibits Src family kinases (SFK), BCR-ABL, platelet-derived growth factor receptor (PDGFR) and c-KIT [37,38] and has previously been shown to inhibit cancer progression as well as viral infection [27,39]. Several studies demonstrated that dasatinib improved the pharmacological response by targeting the phosphorylation of EPHA2 at Ser897. Through inhibition of EPHA2 protein phosphorylation at Ser897, dasatinib inhibits infectivity of *Chlamydia trachomatis* in human epithelial cells [24] and also suppresses IR-induced endothelial cell permeability preventing vascular barrier disruption and leukocyte infiltration into the intestine [40]. Notably, the phosphorylation status of EPHA2 Ser897 is a key determinant of therapeutic response to dasatinib in uterine carcinoma cells [41]. 

**Figure 3:**
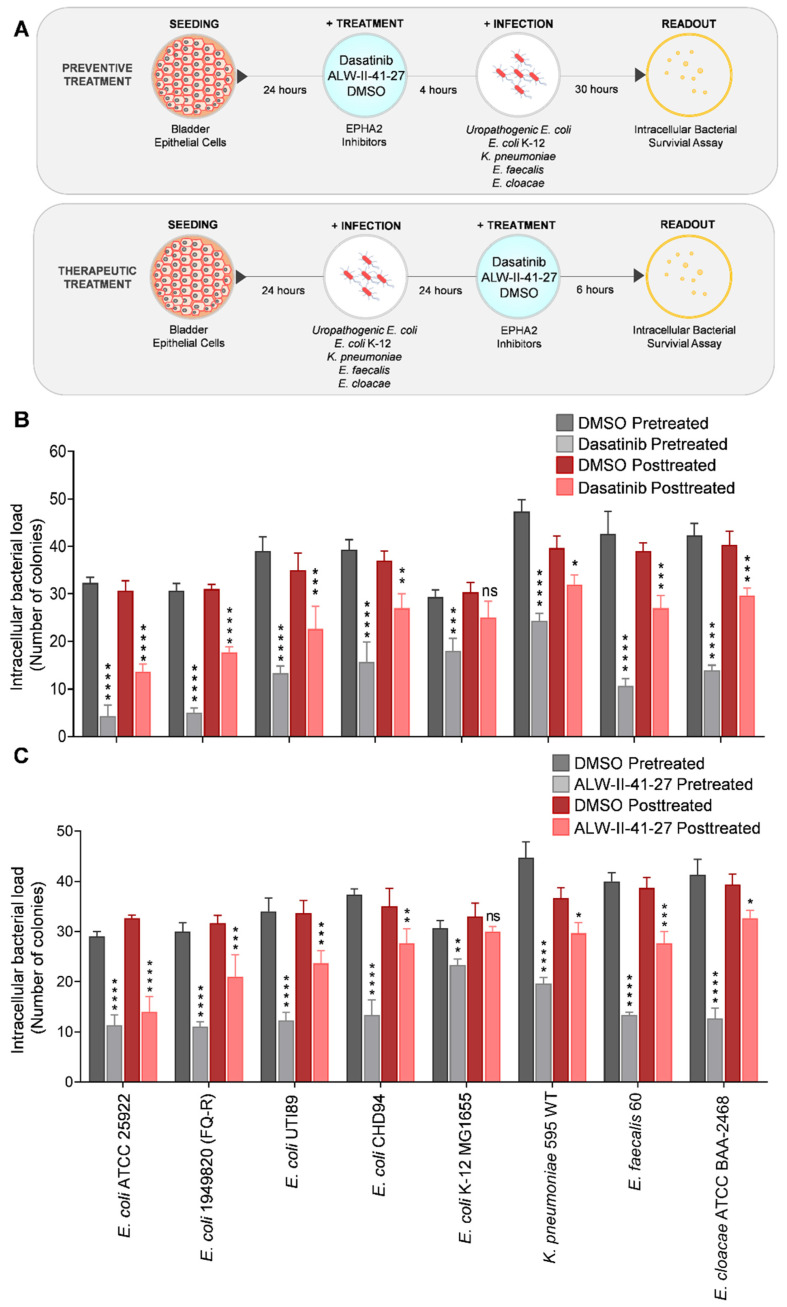
Pharmacological evaluation of EPHA2 inhibition on intracellular bacterial load in human BECs. (**A**) Schematic representation of inhibitor assay performed for figures (**B**,**C**). (**B**) BECs were pretreated with DMSO or dasatinib for 4 h followed by infection with bacteria (MOI, 15–20) for 30 h at 37 °C or BECs were preinfected for 24 h followed by post-treatment with dasatinib for 6 h at 37 °C. Cells were washed three times with 1x PBS. Intracellular bacteria were recovered by means of hypotonic cell lysis as described in materials and methods. Dilutions of the lysates were plated on LB agar and incubated for 24 h at 37 °C. The next day, colonies were counted for each plate. (**C**) BECs were pretreated with DMSO, or ALW-II-41-27 for 4 h followed by infection for 30 h at 37 °C or BECs were preinfected for 24 h followed by post-treatment with ALW-II-41-27 for 6 h at 37 °C. Cells were washed three times with 1x PBS and intracellular bacterial survival assay was performed. (**B**,**C**) Data were presented as mean ± SD of three independent experiments normalized to DMSO pretreated or DMSO post-treated controls, respectively. Statistical analysis was performed using two-way ANOVA followed by Bonferroni multiple comparison test, * *p* < 0.05, ** *p* < 0.01, *** *p* < 0.001, **** *p* < 0.0001, ns-non-significant.

In order to evaluate the impact of tyrosine kinase inhibition on intracellular bacterial survival, the human BECs were subjected to inhibitor treatments before infection (referred to as preventive treatment) and after infection (referred to as therapeutic treatment) with seven uropathogens, as shown in Figure 3A. Preventive treatment of BECs with dasatinib prior to the uropathogen infection significantly reduced the intracellular bacterial load when compared to the DMSO-pretreated BECs (Figure 3B). Further, we next evaluated if dasatinib can reduce the intracellular bacterial load in BECs that were successfully infected with uropathogens for 24 h, as this context mimics the typical clinical infection scenario. Dasatinib treatment caused a significant reduction in the intracellular bacterial load exclusively in the uropathogen-infected BECs (Figure 3B). On the contrary, in the context of dasatinib treatment of BECs pre-infected with non-uropathogenic *E. coli* K-12, there was no significant reduction in intracellular bacterial load. Although we cannot exclude the role of other dasatinib-sensitive kinases such as Src family kinases, BCR-ABL, KIT (a cytokine receptor) and PDGFR [37,38], the drastic reduction in uropathogens infection demonstrates the functional relevance of receptor tyrosine kinases for uropathogens infection and intracellular survival.

In order to affirm the findings from RTK inhibition with dasatinib, we also evaluated a potent and selective small-molecule ATP-competitive inhibitor of EPHA2 called ALW-II-41-27. ALW-II-41-27 was first identified in 2009 [42], which decreased the phosphorylation of EPHA2 at Ser897 in many cancers [43,44,45,46]. The effect of this drug in inducing tumor regression in NSCLCs was first reported in 2014 [44], and since then, the promising effects of this drug have been reported in numerous studies, which has been shown to reduce the tumorigenicity in many cancer cells [47,48,49,50,51,52]. Similar to dasatinib evaluation, both preventive treatment of BECs with ALW-II-41-27 prior to the uropathogen infection and therapeutic treatment of uropathogen-infected BECs with ALW-II-41-27 demonstrated a significant reduction in the intracellular bacterial load exclusively in the uropathogen-infected BECs, but not in BECs pre-infected with non-uropathogenic *E. coli* K-12 (Figure 3C). 

Further, evaluation of EPHA2 inhibitors in a non-malignant cell line HEK293 demonstrated that both preventive and therapeutic pharmacological inhibition of EPHA2 kinase activity by dasatinib and ALW-II-41-27 reduces uropathogen infection comparable to the treatment outcome observed in 5637 bladder cancer epithelial cells (Figure S2A,B). These observations conclude that blocking EPHA2 kinase function by pharmacological EPHA2 inhibition can successfully diminish the intracellular survival of both uropathogens and non-uropathogenic strains, suggesting that interference of EPHA2 signaling is a viable non-antibiotic-based therapeutic strategy for managing recurrent UTIs. 

## 4. Discussion

EPHA2 receptor and its kinase-dependent signaling pathway play a critical role in multiple pathophysiological processes, including development, cancer and pathogen infections. Receptor tyrosine kinases, including EPHA2, have been widely used by many human pathogens for successful infection, intracellular survival and replication within the host cell. In this study, we reported on the identification of EPHA2 as a new host cell factor for successful uropathogenic intracellular infection in the BECs using RNA interference-based post-transcriptional gene silencing and small-molecule inhibitors.

In mouse infection models, intracellular persistence of UPEC within BECs contributes to recurrent UTIs. Approximately 3 to 700 intracellular bacterial communities can be detected in a single bladder within just a few hours after inoculation with UPECs in infected mice [53]. Specific host cell signaling networks are activated by UPECs secreted factors that facilitate the UPECs’ invasion of BECs. In addition to UPECs, other uropathogens also contribute to UTIs. Therefore, understanding the molecular mechanisms underlying the establishment of intracellular bacterial reservoirs is crucial for the development of effective therapeutic strategies to manage persistent and recurrent UTIs. Intracellular uropathogens survive in the host BECs by manipulating several host-signaling pathways. With the progression of infection, among the many host-signaling pathways utilized by uropathogens, we identified EPHA2 as one such signaling platform through which they achieve successful infection. Interference of EPHA2 signaling by knockdown or using inhibitors greatly affected the uropathogenic infection in BECs (Figure 2D). 

Numerous studies proposed the crosstalk between EPHA2-EGFR-PI3K signaling in cancer [54,55]. Lupberger, J. et al., in 2011, identified the RTKs EGFR and EPHA2 as novel host factors for hepatitis C virus entry [27]. Swidergall, M. et al., in 2021, identified Candida albicans virulence factors activating EPHA2-EGFR signaling in oral epithelial cells [56]. Kim, J. et al., in 2022, have shown the Akt-mediated phosphorylation of EPHA2 promoting the interactions of EPHA2 with EGFR in cancer cell lines [57]. PI3K-Akt pathway plays a crucial role in the regulation of cell proliferation, differentiation and survival. Further, manipulating this pathway is an important strategy through which many pathogens promote long-lasting infection. By activating PI3K-Akt signaling, several viruses and bacteria slow down or block apoptosis and prolong the infection. This pathway is usually activated by RTKs, and aberrant activation of RTKs induces multi-fold PI3K activation complicating the disease [58,59,60]. Blocking the PI3K pathway by targeted therapy on RTKs with tyrosine kinases inhibitors has achieved great progress in cancer [61]. In a recent study by Kim W.J. et al., RTK-EGFR mediates UPEC-induced p-Akt promoting bacterial invasion [11]. Many studies previously identified EPHA2 as being upstream signaling of PI3K-Akt in certain pathogen infections [23,24]; therefore, we hypothesized that uropathogens might also utilize EPHA2 signaling. Since our EPHA2 knockdown study convincingly reduced the uropathogens infection, we made use of the approved EPHA2 inhibitors such as dasatinib and ALW-II-41-27. 

Dasatinib has previously been shown to suppress several infections, such as chlamydial infection [24], reduce the growth of intracellular Mycobacterium tuberculosis [62] and inhibit HIV replication [63] and HCV infection [27]. Similarly, ALW-II-41-27, an Eph receptor tyrosine kinase inhibitor with an IC50 of 11 nM for EPHA2, was effective in inhibiting oxidative stress and inflammation in *Trichinella spiralis*-infected mice [48]. However, we could not find any study about these inhibitors in uropathogens infection. Since these inhibitors affect a majority of signaling pathways, we hypothesized it could also affect the uropathogens infection, and we could demonstrate in our study that both the inhibitors significantly reduced the intracellular infection in an in vitro BECs culture model. 

It is evident from our results that the intracellular survival of uropathogen is dependent on EPHA2 signaling, as inhibition of EPHA2 signaling pre- and post-infection significantly reduced the infection in BECs (Figure 3B,C). Importantly, efficient binding to and entry into host cells is a crucial step for many pathogens to establish successful infection. Since our results demonstrate that the pretreatment of epithelial cells with RNA interference or inhibitor targeting EPHA2 prior to infection significantly reduced intracellular bacterial survival, the EPHA2 receptor can be a critical factor during the early phase of uropathogen infection. Further investigation is required to validate if EPHA2 can serve as an entry receptor during early infection. It will also be noteworthy to identify the bacterial factors that mediate the interaction with EPHA2, thereby promoting its invasion process. Although both EPHA2-specific RNA interference and small-molecule compounds have shown promising impacts on the reduction in bacterial survival, both dasatinib and ALW-II-41-27 are nevertheless multi-kinase inhibitors, thus reiterating the need for the development of selective small-molecules targeting specific RTKs such as EPHA2 to evaluate their translational potential, while circumventing unintended off-target effects.

**Figure 4:**
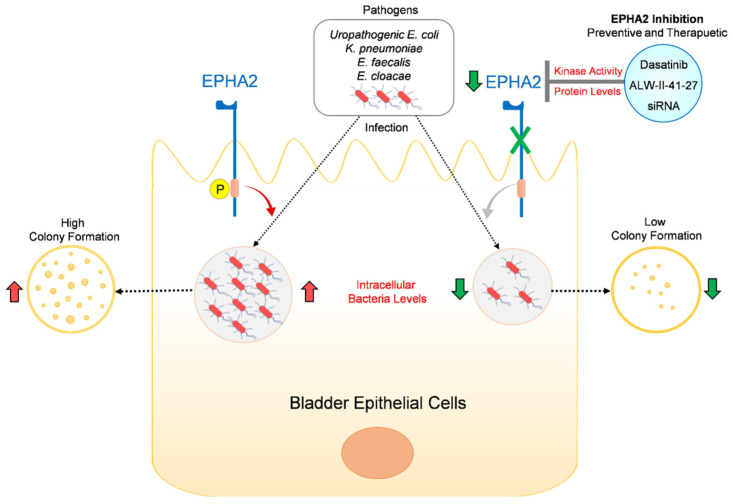
Summary figure. Targeting EPHA2 expression by gene-specific RNA interference or blocking the kinase activity of EPHA2 by pharmacological compounds such as dasatinib or ALW-II-41-27 in a preventive or therapeutic treatment approach in human BECs caused significant reduction in the intracellular bacterial survival as determined by colony formation. Our findings conclude that interference of EPHA2 signaling is a viable non-antibiotic-based therapeutic strategy for managing recurrent UTIs caused by wide range of uropathogens.

In summary, we demonstrated that targeting EPHA2 significantly reduced intracellular uropathogenic survival in BECs (Figure 4), which can be further extended to pharmacology studies for in vivo evaluation of small-molecules selectively targeting EPHA2 and repurposing potent multi-kinase inhibitors targeting relevant RTKs to design non-antibiotic therapeutic approaches to ultimately alleviate recurrent UTIs.

## Data Availability

All data generated and analyzed during this study are included in this published article. Raw data supporting the findings of this study are available from the corresponding author on request.

## Supplementary Materials

The following are available online at https://www.mdpi.com/article/10.3390/pathogens11101176/s1, Figure S1: Characterization of EPHA2 receptor levels upon infection with different uropathogens. Figure S2: Pharmacological evaluation of EPHA2 inhibition on intracellular bacterial load in HEK293 cells
