# Supplementary material for: Targeting Host Tyrosine Kinase Receptor EPHA2 Signaling Affects Uropathogen Infection in Human Bladder Epithelial Cells
Source: Pathogens. 2022 Oct 12;11(10):1176. doi: 10.3390/pathogens11101176 (PMC9607038; doi:10.3390/pathogens11101176)
Supplement: Supplementary file 1 [file pathogens-11-01176-s001.zip › pathogens-1904981-supplementary.pdf]

pathogens

MDPI

Article

Targeting host tyrosine kinase receptor EPHA2 signaling affects uropathogen infection in human bladder epithelial cells

Prema S. Prakash *, Alexander Kruse, Christian Vogel, Undraga Schagdarsurengin and Florian Wagenlehner

Clinic for Urology, Pediatric Urology and Andrology, Justus-Liebig-University, Giessen, Germany

* Correspondence: premaprakash17@gmail.com

Supplementary Materials:

Fig S1A

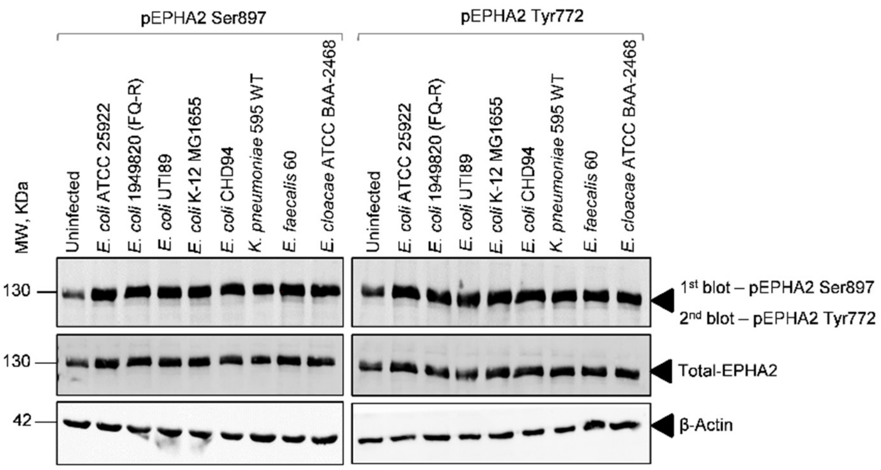

Fig S1B

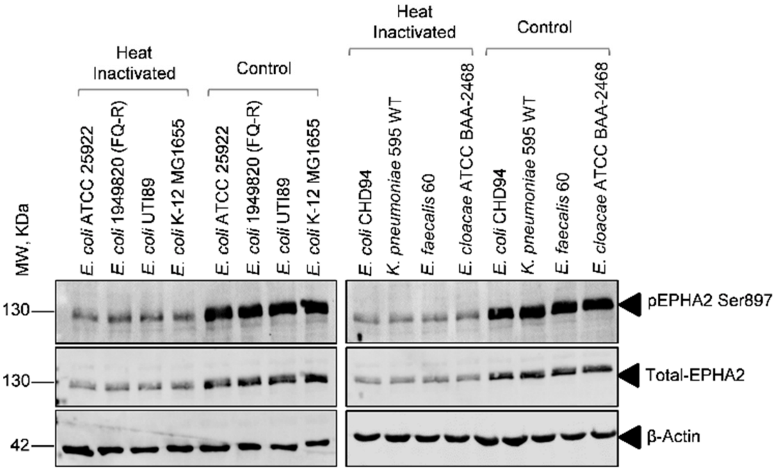

Figure S1. Characterization of EPHA2 receptor levels upon infection with different uropathogens. **(A)** Cells infected with bacteria were harvested for western blot analysis to detect the serine and tyrosine phosphorylation status of EPHA2 upon different uropathogens infection. Protein quantity was equally loaded for each sample in both blots. Blots were placed adjacently and imaged at the same time. **(B)** BECs were infected with heat inactivated bacteria (70 °C, 20 min) or control without any inactivation for 30 h at 37 °C. Medium was removed and the cells were washed three times with 1x ice cold PBS. Cells were then harvested and subjected to western blot analysis.

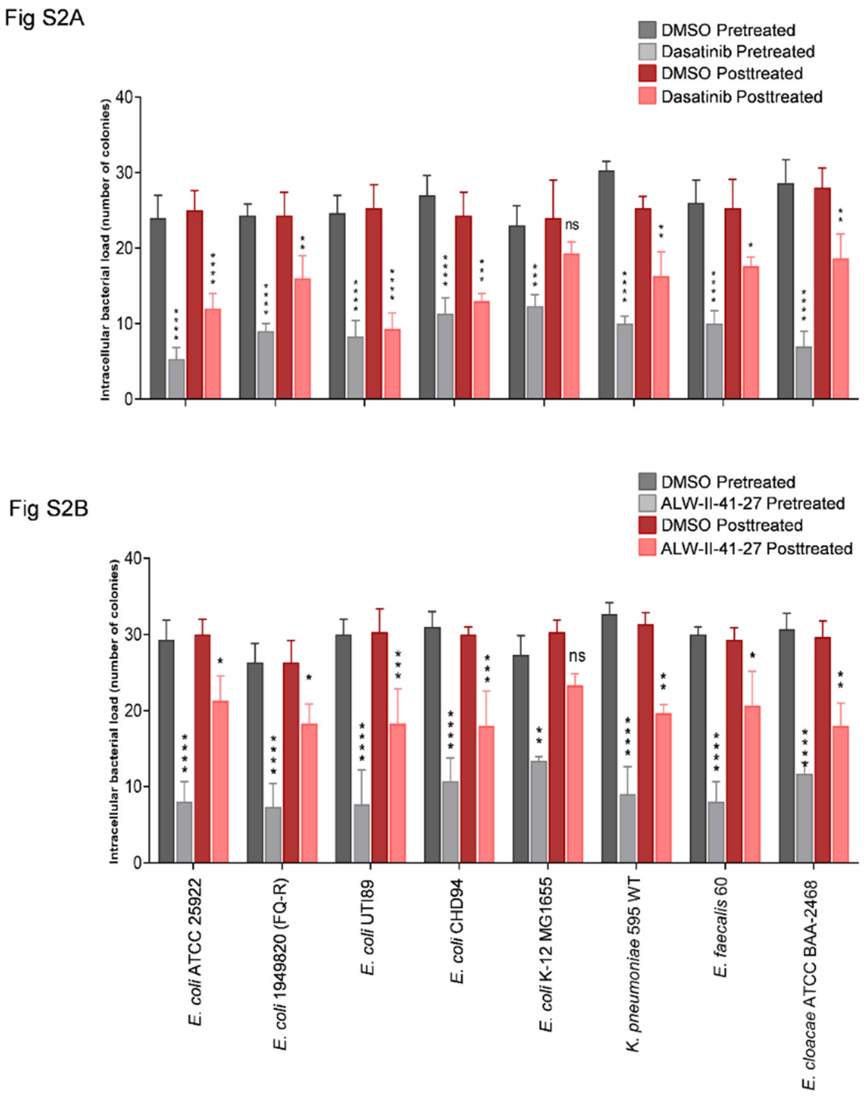

Figure S2. Pharmacological evaluation of EPHA2 inhibition on intracellular bacterial load in HEK293 cells. **(A)** HEK293 cells were pretreated with DMSO or dasatinib for 4 h followed by infection with bacteria (MOI, 15-20) for 30 h at 37°C or HEK293 cells were preinfected for 24 h followed by post-treatment with dasatinib for 6 h at 37 °C. Cells were washed three times with 1x PBS. Intracellular bacteria were recovered by means of hypotonic cell lysis as described in materials and methods. Dilutions of the lysates were plated on LB agar and incubated for 24 h at 37 °C. Next day, colonies were counted for each plate. **(B)** HEK293 cells were pretreated with DMSO, or ALW-II-41-27 for 4 h followed by infection for 30 h at 37 °C or cells were preinfected for 24 h followed by post-treatment with ALW-II-41-27 for 6 h at 37°C. Cells were washed three times with 1x PBS and intracellular bacterial survival assay was performed. (A) and (B) Data were presented as mean ± SD of three independent experiments normalized to DMSO pretreated or DMSO posttreated controls respectively. Statistical analysis was performed using two-way Anova followed by Bonferroni multiple comparison test, *$p < 0.05$, **$p < 0.01$, ***$p < 0.001$, ****$p < 0.0001$., ns-non-significant.
